# Assessment of university students’ earthquake coping strategies using artificial intelligence methods

**DOI:** 10.1038/s41598-025-17555-4

**Published:** 2025-08-29

**Authors:** Suleyman Alpaslan Sulak, Nigmet Koklu

**Affiliations:** 1 Ahmet Kelesoglu Educational Faculty, Necmettin Erbakan University, Konya, Türkiye; 2 Vocational School of Technical Sciences, Konya Technical University, Konya, Türkiye

**Keywords:** Coping with earthquake stress scale (CESS), Machine learning algorithms, Logistic regression. bagging, Random forest, Natural hazards, Engineering, Mathematics and computing

## Abstract

Earthquakes are one of the most destructive natural disasters that pose a serious threat to human life and infrastructure worldwide. The aim of this study is to evaluate the coping strategies of adult individuals in Turkey regarding earthquake stress using artificial intelligence-based methods. The data was collected from 858 university students living in Turkey during January, February, and March 2024. A dataset was created using the ‘Coping Scale for Earthquake Stress.’ Prediction models were established using artificial intelligence algorithms such as Logistic Regression (LR), Bagging, and Random Forest (RF) based on information from 24 variables. The cross-validation method was applied during model training. The Logistic Regression algorithm achieved the highest accuracy rate of 98.60%, while the Bagging algorithm demonstrated the lowest performance with an accuracy rate of 79.95%. The Random Forest algorithm showed moderate performance with an accuracy rate of 85.89%. The findings provide important insights into the coping strategies of the community regarding earthquake stress. This study is expected to contribute significantly to areas such as disaster management, psychology, public health, and community resilience.

## Introduction

Earthquakes are natural disasters that can deeply affect societies with their destructive power. Strong tremors can cause buildings to collapse, infrastructure to be destroyed and landslides to occur. This destruction leads to many casualties, injuries and homelessness. The intensity, magnitude and effects of earthquakes vary depending on factors such as the epicenter, depth and local construction conditions. The most important measure against earthquakes is to build earthquake-resistant structures and raise public awareness.

In line with these efforts, advanced technologies such as artificial intelligence are increasingly being used in areas such as earthquake risk analysis, early warning systems and damage assessment. Emergency preparedness, building earthquake-resilient structures, raising public awareness and preparing emergency plans play an important role in reducing the devastating effects of earthquakes.

The aim of our research is to determine coping strategies with earthquake stress using artificial intelligence techniques. Earthquake stress is characterized by intense emotional, physical and psychological reactions during and after an earthquake. Sudden and uncontrollable events such as earthquakes evoke a deep sense of fear, anxiety and uncertainty in individuals. This stress can negatively affect people’s daily lives both immediately and in the long term. Physical symptoms include heart palpitations, sweating, nausea and headaches^1^, while emotionally intense feelings of anxiety, anger, and depression^2^ are common. Psychologically, long-term effects such as post-traumatic stress disorder (PTSD), nightmares and a constant state of alertness can occur^3^. These responses are the result of the body trying to cope with stress and can affect the overall health of individuals in both the short and long term.

Earthquake stress is quite common, especially in societies living in earthquake zones. It can affect not only the mental health of individuals but also societal resilience^4^. Increased stress and anxiety can complicate post-disaster recovery^5^. Therefore, it is of great importance to recognize earthquake stress and develop appropriate strategies to cope with it.

Various personal strategies can be applied to cope with earthquake stress. Physical activity, exercise improves mood by reducing stress hormones^6^. Activities such as walking, jogging or yoga can be effective in coping with stress^7^. Relaxation techniques, such as deep breathing, meditation and mindfulness, calm the mind and reduce stress levels^8^. In addition, regular sleep, a balanced diet and adequate water consumption support overall physical and mental health, which makes it easier to cope with stress^9^.

Social support plays an important role in managing earthquake stress. Talking to family, friends or support groups can provide emotional support and reduce feelings of loneliness. Utilizing resources such as community centers or post-disaster support programs can strengthen the social support network and increase social solidarity^10,11^.

Professional support is also an effective coping strategy. Psychotherapy can address emotional and psychological trauma through individual or group therapies. Seeing specialists can help to cope with long-term psychological effects such as PTSD. In addition, pre-earthquake preparation and planning, earthquake kit preparation and family emergency plans can reduce anxiety and increase a sense of safety^12^. Information and education, knowledge about earthquake safety and first aid can increase the sense of control and facilitate coping with stress^13^.

Rouet-Leduc et al. applied artificial intelligence methods to data sets obtained from earthquake experiments in the laboratory. In this study, it is shown that artificial intelligence methods can predict when a fault will rupture by listening to acoustic signals emitted from a laboratory fault. They suggest that applying their approach to real seismic data could lead to advances in identifying unknown signals, gaining new insights into fault physics, and predicting fault rupture times^14^. Xie et al. examine the progress and challenges in the application of artificial intelligence methods in earthquake engineering. Their review shows the extent to which artificial intelligence methods have been applied in four main areas such as seismic hazard analysis, system identification and damage detection, seismic vulnerability assessment, and structural control for earthquake mitigation^15^. Murtanwara et al. aim to compare the performance of artificial intelligence algorithms for predicting earthquakes in Indonesia in the medium and long term. The dataset they used includes medium and long-term earthquake data collected from 2 local government agencies and 8 international sources. Within the scope of the study, Support Vector Machine (SVM) showed the lowest error rate with 0.751. SVM was followed by Logistic Regression with 0.777, while Naive Bayes had the highest error rate with 0.922^16^. Ridzwan aims to introduce a method for the prediction and modeling of earthquake impacts. The dataset used in the study consists of past earthquake records, ground motion data and relevant environmental factors in a given region. Most of the models they examined focused on predicting earthquake magnitude, trend and occurrence^17^. Hussain et al., emphasize three main factors that increase the magnitude of the Kahramanmaraş earthquake. These are exposure, corruption and poverty. Deficiencies in the implementation of building codes, the “zoning peace” announced in 2018, and high poverty rates in the region stand out as factors that increase the impact of the earthquake. This study emphasizes that in order to reduce disaster risk, it is not only sufficient to understand the seismic hazard, but also the social and built environments should be managed effectively^18^.

Current research on earthquake-induced stress primarily focuses on psychological, social, and demographic factors, often relying on traditional statistical analysis methods. However, studies utilizing artificial intelligence-based approaches to analyze individuals’ coping strategies in disaster contexts remain quite limited. Notably, university students —who are often separated from family support systems and live in densely populated urban areas— represent a high-risk group that is frequently underrepresented in the literature.

This study addresses this gap by focusing specifically on university students and analyzing their earthquake coping strategies using machine learning algorithms. The integration of artificial intelligence techniques enables the identification of behavioral patterns related to coping, offering a data-driven and innovative method for assessing psychological resilience and preparedness.

Structured from an interdisciplinary perspective, this research aims to bridge psychology and artificial intelligence to support the development of more predictive, objective, and scalable assessment tools for post-disaster mental health interventions.

## Materials and methods

**Fig. 1:**
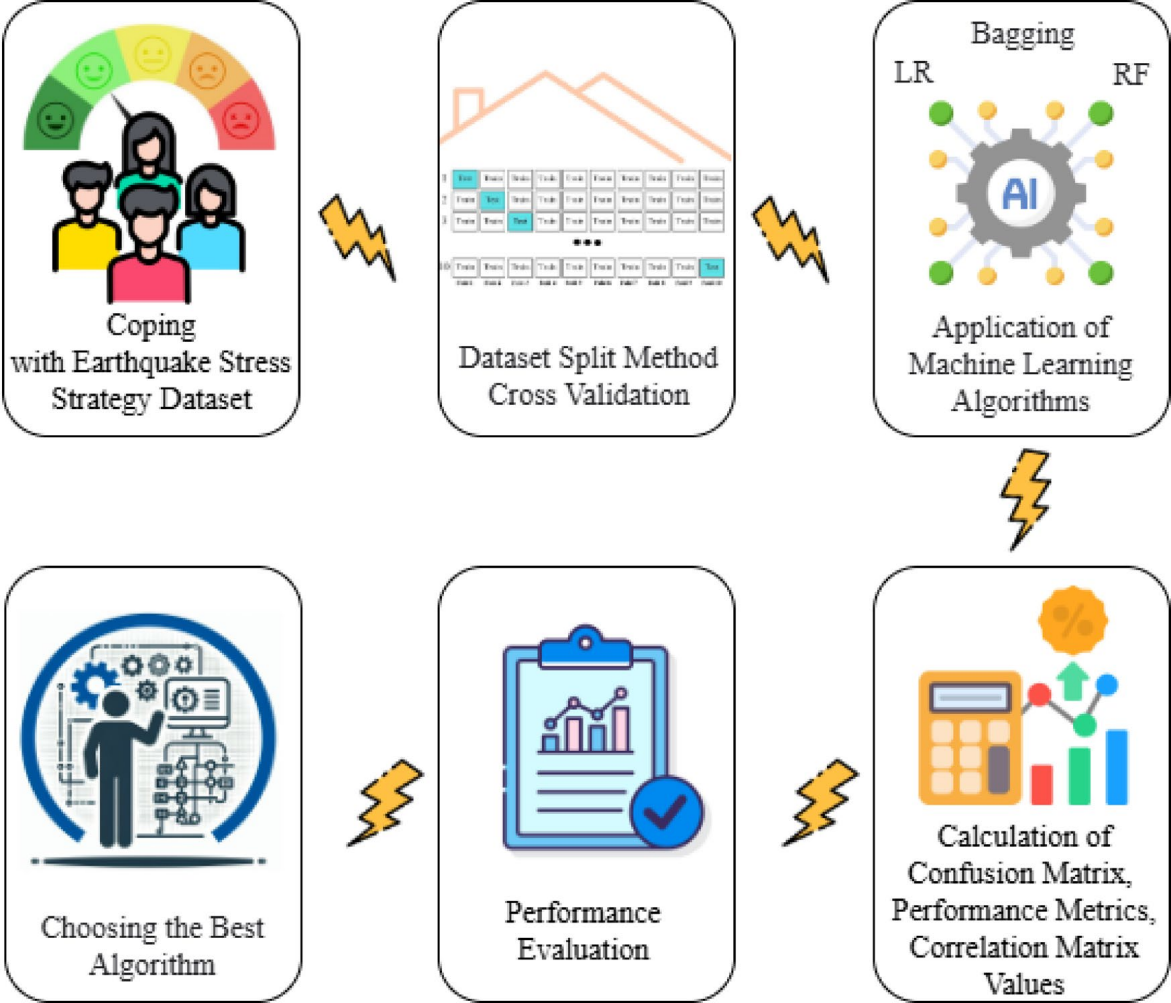
Flow chart of the study.

In this section; information about Coping with Earthquake Stress Strategy Dataset, Confusion Matrix and Performance Metrics, Matthews Correlation Coefficient, Chi-Square Test, Correlation Matrix, Cross Validation, Artificial Intelligence Models, Logistic Regression, Bagging and Random Forest are given. Flow chart of the study is shown in Fig. 1. All methods were performed in accordance with the relevant guidelines and regulations, and the study was approved by Ethics Committee for Social and Behavioral Sciences at Necmettin Erbakan University, in line with the Declaration of Helsinki.

### Coping with earthquake stress strategy dataset (CESS dataset)

In the study, the Scale of Coping Strategies with Earthquake Stress developed by Yondem and Eren was used^19^. Earthquake Stress Coping Strategies Scale was applied to 858 people. The data were collected electronically and made available for analysis. The created data set consists of 24 variables in total. These extracted features are transferred to the dataset. Using the cross-validation method, the trained data are directed to classification models. The trained algorithms provide output according to the determined classes.

**Fig. 2:**
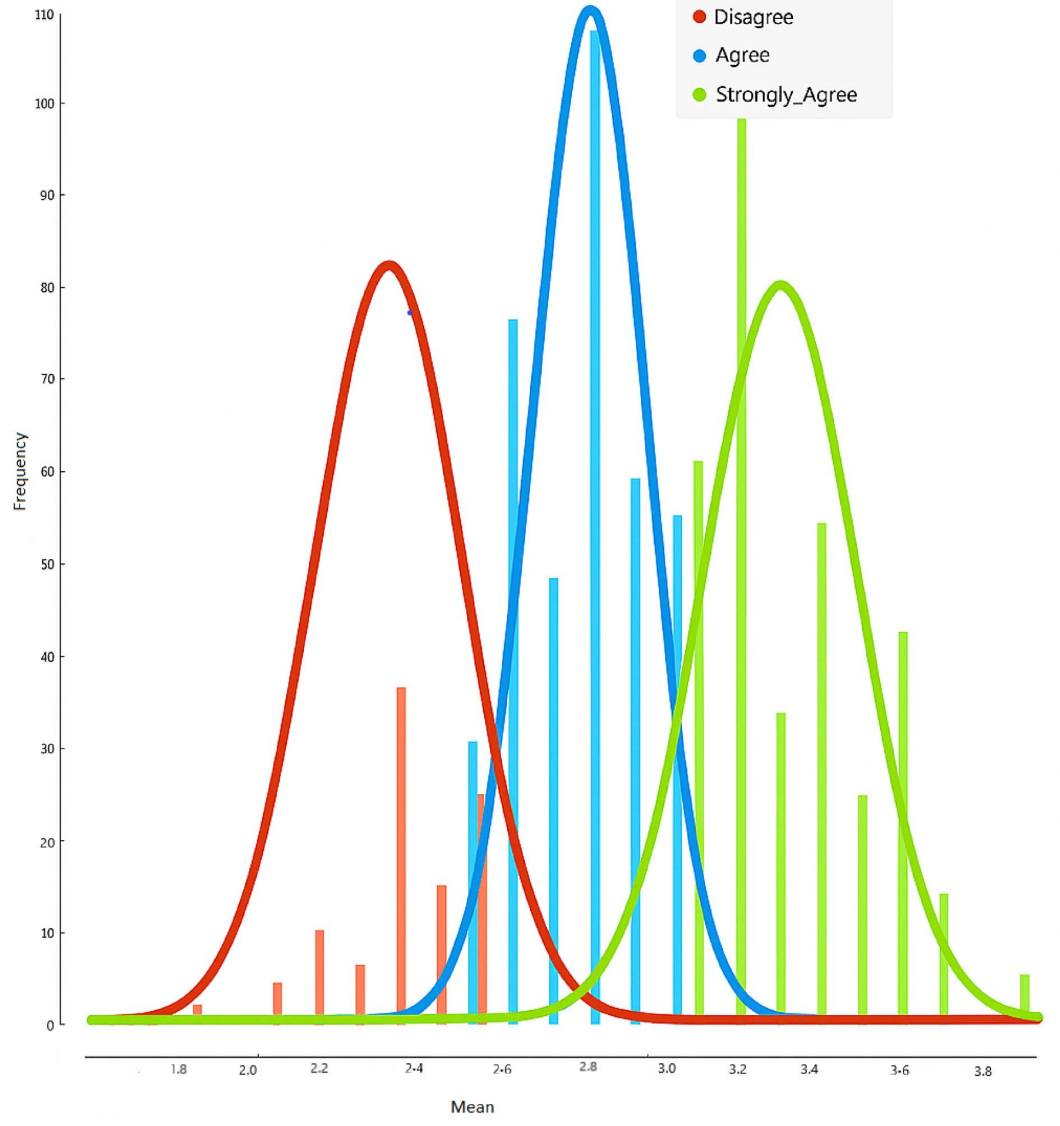
Threshold-based frequency distributions.

In the data set creation stages, demographic questions were first asked and then a scale consisting of 16 questions was used. Informed consent was obtained from all participants prior to their involvement in the study. Data were collected from 858 university students between 19.01.2024 and 07.03.2024. In total, a data set consisting of 24 variables was obtained. The 24 variables are shown in Table 1. The dataset in Table 1 includes both categorical variables (e.g., gender, residence type) and ordinal Likert-scale items. Categorical variables were processed using label encoding to convert them into numerical values. The Likert-scale items were treated as ordinal data to preserve their inherent order. No normalization or scaling was applied to these variables. Class labels were determined based on the average scores participants received from the 16-item CESS scale, using threshold values as follows: 1.00–2.50 = Disagree; 2.51–3.00 = Agree; 3.01–5.00 = Strongly Agree. While setting these thresholds, the frequency distribution of the mean CESS scores was examined. It was observed that the frequencies clustered within these defined intervals. This distribution is illustrated in Fig. 2. Therefore, the data were categorized into three distinct classes for classification purposes. This classification approach reflects the ordinal nature of Likert-type data. Standard classifiers were selected over ordinal-specific ones as the clustered distributions enabled effective nominal separation, as evidenced by high MCC values. The results obtained from these variables consist of 3 classes. These are:

**Table 1:** Coping with earthquake stress strategy dataset Variables.

Attributes	Values
Gender	1-Male; 2-Feale
Age	Values in integer
Grade	1; 2; 3; 4; 5
Have_you_encountered_an_earthquake_before	1-Yes; 2-No
Living_Place	1-Village; 2-Town; 3-District; 4-City; 5-Metropolitan
Building_Floor	1-Detached House; 2–2/3 floor; 3–4/5 floor; 4–6 + floor
Year_of_Construction	1–1/5 year; 2-6-10 year; 3–11/15 year; 4–16/20 year;5–21 + year
Residence_Type	1-Own; 2-Rental
1) I share what I experience with my family or friends.	1-Strongly disagree; 2-Disagree; 3-Rarely; 4-Agree; 5-Strongly agree
2) I entrust myself to God.	1-Strongly disagree; 2-Disagree; 3-Rarely; 4-Agree; 5-Strongly agree
3) I try to keep my feelings to myself.	1-Strongly disagree; 2-Disagree; 3-Rarely; 4-Agree; 5-Strongly agree
4) I talk to someone who can cope with this problem better.	1-Strongly disagree; 2-Disagree; 3-Rarely; 4-Agree; 5-Strongly agree
5) Despite all the negativity related to the earthquake, I keep fighting.	1-Strongly disagree; 2-Disagree; 3-Rarely; 4-Agree; 5-Strongly agree
6) I share my feelings and fears with my friends or family.	1-Strongly disagree; 2-Disagree; 3-Rarely; 4-Agree; 5-Strongly agree
7) I prefer not to talk about my fears and anxieties.	1-Strongly disagree; 2-Disagree; 3-Rarely; 4-Agree; 5-Strongly agree
8) I think that death is inevitable.	1-Strongly disagree; 2-Disagree; 3-Rarely; 4-Agree; 5-Strongly agree
9) I try to find comfort through prayer.	1-Strongly disagree; 2-Disagree; 3-Rarely; 4-Agree; 5-Strongly agree
10) I believe in fate and that it cannot be changed.	1-Strongly disagree; 2-Disagree; 3-Rarely; 4-Agree; 5-Strongly agree
11) I fulfill my religious duties more often.	1-Strongly disagree; 2-Disagree; 3-Rarely; 4-Agree; 5-Strongly agree
12) I accept what has happened as an experience.	1-Strongly disagree; 2-Disagree; 3-Rarely; 4-Agree; 5-Strongly agree
13) I try to be more optimistic about life.	1-Strongly disagree; 2-Disagree; 3-Rarely; 4-Agree; 5-Strongly agree
14) I try to think positively.	1-Strongly disagree; 2-Disagree; 3-Rarely; 4-Agree; 5-Strongly agree
15) I believe that giving myself time will be helpful.	1-Strongly disagree; 2-Disagree; 3-Rarely; 4-Agree; 5-Strongly agree
16) I try not to make a big deal out of things.	1-Strongly disagree; 2-Disagree; 3-Rarely; 4-Agree; 5-Strongly agree
Coping with Earthquake Stress Strategy Class	1- Disagree (This person disagrees with the Coping with Earthquake Stress Strategy) 2- Agree (This person agrees with the Coping with Earthquake Stress Strategy) 3- Strongly agree (This person strongly agrees with the Coping with Earthquake Stress Strategy)

**Table 2:** Sample confusion matrix notation and Explanations.

	Predicted					
Actual	Confusion Matrix	Disagree	Agree	Strongly Agree	**TP** The number of correctly predicted disagree	*FN* The number of predicted agree but actually disagree
	Disagree	T_1_	F_12_	F_13_		
	Agree	F_21_	T_2_	F_23_	*FP* The number of predicted disagree but actually agree	**TN** The number of correctly predicted agree
	Strongly Agree	F_31_	F_32_	T_3_

- Disagree (This person disagrees with the Coping with Earthquake Stress Strategy).
- Agree (This person agrees with the Coping with Earthquake Stress Strategy).
- Strongly agree (This person strongly agrees with the Coping with Earthquake Stress Strategy).

The Logistic Regression algorithm was chosen as a fundamental method due to its high interpretability and suitability for multi-class classification problems. Ensemble algorithms such as Random Forest and Bagging were preferred for their robustness in handling the complex and non-linear relationships often observed in psychological data, their resistance to overfitting, and their ability to achieve high accuracy. These ensemble methods enhance model generalizability by reducing variance.

### Confusion matrix and performance metrics

Confusion Matrix and Performance Metrics are basic tools used to evaluate and compare the performance of artificial intelligence algorithms. These tools are used to determine how accurately the model works in classification processes and prediction processes^20^.

It is a table used to evaluate the performance of artificial intelligence algorithms. The table provides 4 pieces of information: TP, TN, FP and FN. According to this information, it can be observed which types of errors the algorithm makes and in which classes it is successful^21–23^. The sample confusion matrix representation and explanations in this study are presented in Table 2. The calculation of TP, TN, FP and FN values are presented in Table 3.

**Table 3:** The calculation of TP, TN, FP and FN values.

$TP_{1} = T_{1}$	$TP_{2} = T_{2}$	$TP_{3} = T_{3}$
$TN_{1} = T_{2} + T_{3} + F_{{23}} + F_{{32}}$	$TN_{2} = T_{1} + T_{3} + F_{{13}} + F_{{31}}$	$TN_{3} = T_{1} + T_{2} + F_{{12}} + F_{{21}}$
$FP_{1} = F_{{21}} + F_{{31}}$	$FP_{2} = F_{{12}} + F_{{32}}$	$FP_{3} = F_{{13}} + F_{{23}}$
$FN_{1} = F_{{12}} + F_{{13}}$	$FN_{2} = F_{{21}} + F_{{23}}$	$FN_{3} = F_{{31}} + F_{{32}}$

It consists of statistical metrics used in artificial intelligence methods and data science to measure how well the model performs^24^. These metrics are used to evaluate the accuracy, precision, errors or overall performance of the model^25,26^. In this study, accuracy, precision, recall and F1-score metrics are used. The descriptions and formulas of the metrics used are given in Table 4.

**Table 4:** Descriptions and formulas of performance metrics and Matthews correlation Coefficient.

Metrics	Description	Formula	
Accuracy	It is used to calculate the average accuracy values of classes.	$\:\frac{{\sum\:}_{i=1}^{1}\frac{{tp}_{i}+{tn}_{i}}{{tp}_{i}+{fn}_{i}+{fp}_{i}+{tn}_{i}}}{l\:}$	(1)
Precision	It is used to calculate the average precision values f classes.	$\:\frac{{\sum\:}_{i=1}^{1}\frac{{tp}_{i}}{{tp}_{i}+{fp}_{i}}}{l}$	(2)
Recall	It is used to calculate the average recall values of classes.	$\:\frac{{\sum\:}_{i=1}^{1}\frac{{tp}_{i}}{{tp}_{i}+{fn}_{i}}}{l}$	(3)
F1-Score	It is used to get the harmonic average of Precision and Recall values.	$\:\frac{2*\:\frac{{\sum\:}_{i=1}^{1}\frac{{tp}_{i}}{{tp}_{i}+{fp}_{i}}}{l}*\:\frac{{\sum\:}_{i=1}^{1}\frac{{tp}_{i}}{{tp}_{i}+{fn}_{i}}}{l}}{\frac{{\sum\:}_{i=1}^{1}\frac{{tp}_{i}}{{tp}_{i}+{fp}_{i}}}{l}+\:\frac{{\sum\:}_{i=1}^{1}\frac{{tp}_{i}}{{tp}_{i}+{fn}_{i}}}{l}}$	(4)
MCC	It is used evaluates classification performance by considering true and false positives and negatives, functioning like a correlation coefficient between predicted and actual classes.	$\:\frac{c.s-{\sum\:}_{k}^{}{p}_{k}{t}_{k}\:}{\sqrt{({c}^{2}-{\sum\:}_{k}^{}{{p}_{k}^{2}}_{}\left)\right({c}^{2}-{\sum\:}_{k}^{}{{t}_{k}^{2}}_{})}}$	(5)

Four main performance metrics are used to evaluate the success of the model. Accuracy represents the ratio of correct predictions made by the model to the total number of predictions. Precision indicates the proportion of correctly predicted positive results among all positive predictions. Recall shows how well the model identifies the actual positive cases. The F1-Score is the balanced average of precision and recall, used to measure the overall performance of the model. The formulas for these metrics are presented in Table 4.

### Matthews correlation coefficient (MCC)

Matthews Correlation Coefficient (MCC) is a robust and comprehensive statistical measure used to evaluate the performance of classification models. It is especially valued for providing reliable results on imbalanced datasets. Unlike basic metrics such as accuracy, MCC takes into account true positives, true negatives, false positives, and false negatives simultaneously, offering a more nuanced assessment^27^. Its values range from − 1 to + 1, where + 1 indicates perfect classification, 0 corresponds to random guessing, and − 1 signifies complete misclassification. While commonly applied in binary classification problems, MCC also has generalized versions suitable for multi-class classification. By reflecting the overall balance of performance across all classes, MCC provides a fair and holistic evaluation of a model. In this study, the MCC values calculated for Logistic Regression, Random Forest, and Bagging algorithms were high, highlighting their accuracy and reliability as key indicators of model performance. The MCC values are shown in tables for algorithms.

### Chi-square test (χ²)

Chi-Square Test (χ²): The Chi-Square test is a statistical method used in classification problems to determine whether a feature has a significant association with target classes. It is especially preferred when working with categorical data. This test helps identify the degree to which each feature is related to the classes, aiming to determine which features truly contribute to the classification process^28^.

The Chi-Square test can be applied directly to the data without the need for model training^29^. This allows for identifying significant variables independently of any specific model. As a result of the test, each feature receives a score that reflects its ability to discriminate between classes. Features with high scores are considered more important, while those with low scores can be deemed unnecessary and removed from the dataset.

This approach not only speeds up the model training process but can also improve accuracy. Additionally, its fast execution on large datasets is a significant advantage. The Chi-Square test is particularly effective and reliable as a preliminary analysis tool when working with categorical data or numerical data that can be discretized into categories.

As a result of the Chi-Square test, the three features contributing most significantly to the classification process were identified as CESS_10 (“I believe in fate and that it cannot be changed,” χ² = 130.674), CESS_13 (“I try to be more optimistic about life,” χ² = 85.521), and CESS_11 (“I fulfill my religious duties more often,” χ² = 79.574) (Table 5).

**Table 5:** Results of χ² variables.

Items	χ²
CESS_10	130.674
CESS_13	85.521
CESS_11	79.577
CESS_14	76.778
CESS_9	74.783
CESS_8	66.001
CESS_2	54,785
CESS_12	47.237
CESS_4	44.261
CESS_15	42.212
CESS_16	40.513
CESS_6	27.073
CESS_5	24.313
CESS_7	18.340
CESS_1	10.758
Residence_Type	5.131
Living_Place	2.910
CESS_3	2.181
Gender	1.985
Grade	1.274
Building_Floor	1.046
Have_you_encountered_an_earthquake_before	0.634
Year_of_Construction	0.046
Age	0.038

### Correlation matrix

A correlation matrix is a symmetric square matrix that quantitatively represents the relationships between variables in a multivariate data set. This matrix contains the Pearson correlation coefficients between each pair of variables, ranging from − 1 to + 1, indicating the direction and strength of the relationship. The diagonal elements of the matrix are always 1 because the correlation of a variable with itself is strictly positive. The correlation matrix is used as a fundamental tool in multidimensional data analysis, factor analysis and various statistical modeling techniques, providing researchers with critical information in understanding the complex relationships between variables^30,31^.

−1: It represents a strong and negative correlation between two variables.

0: It represents the absence of a relationship between two variables.

1: It represents a strong and positive correlation between two variables

### Cross validation

**Fig. 3:**
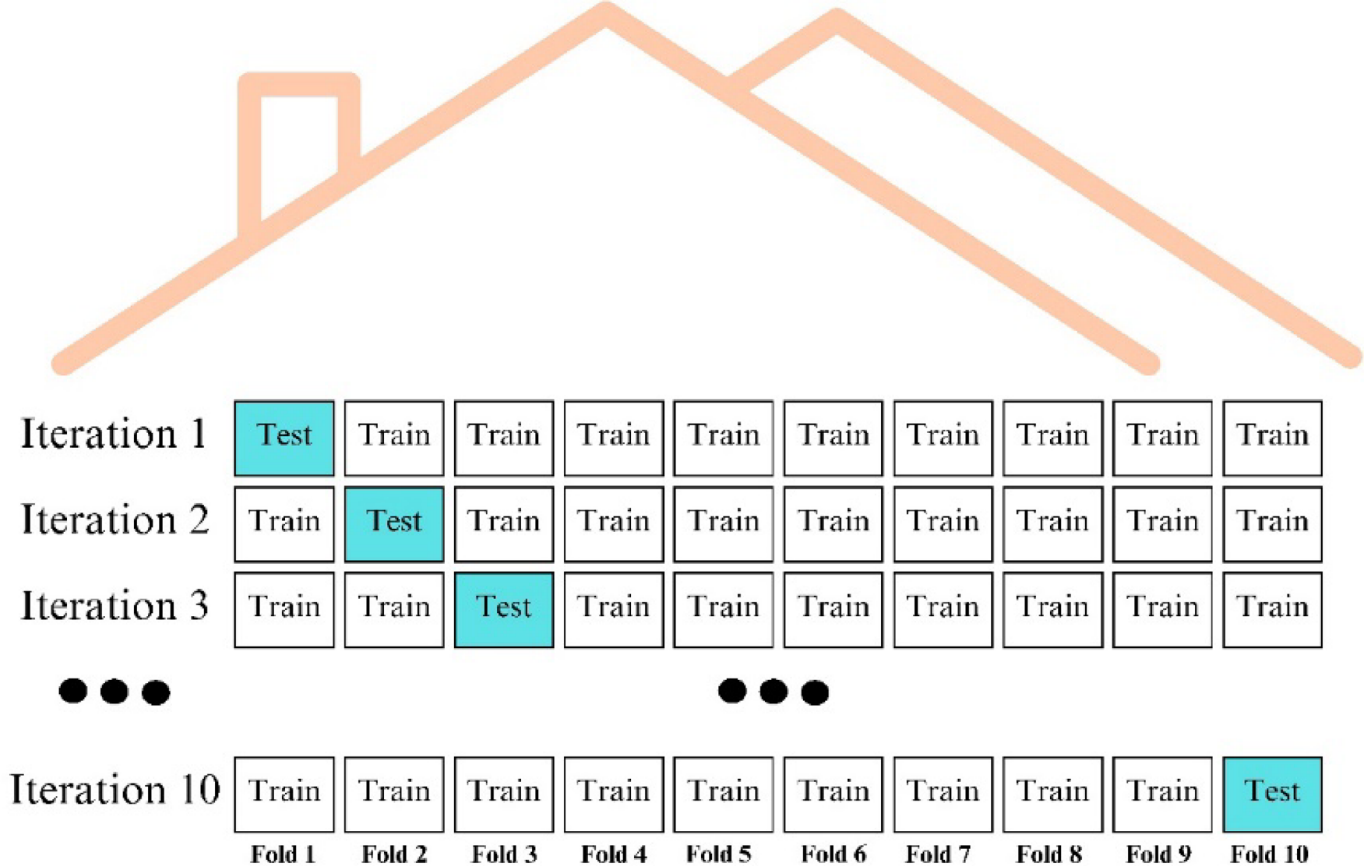
Cross Validation Method.

Cross validation is a statistical resampling method used to evaluate the performance of an artificial intelligence model on unseen data as objectively and accurately as possible. In this method, the data set is divided into k parts and one part at a time is used as a test set. The rest are used as the training set. This process is repeated for all parts and the performance of the model is measured at each iteration. The overall performance of the model is determined by averaging the results. This approach helps to understand how the model performs on different data subsets and helps to obtain more reliable results^22,32,33^. Cross validation is presented in Fig. 3. In this study, stratified folds were intentionally not used due to the relatively small size and clustered nature of the dataset, which could lead to overly homogeneous folds and affect model training. Instead, we used random folds to better reflect the natural variability of the data.

### Artificial intelligence models

Artificial intelligence models are computer systems designed to perform various tasks. These models try to mimic human intelligence and demonstrate advanced capabilities in certain areas^34^. Logistic Regression, bagging and random forest algorithms were used in this study.

#### Logistic regression

Logistic regression is a statistical method and artificial intelligence algorithm used in classification problems. It is mainly used to model the relationship between independent variables and a categorical dependent variable. It uses the logistic function to transform the input variables into a probability value between 0 and 1. This probability usually represents the probability of an event occurring or belonging to a class. Logistic regression is widely used, especially in binary classification problems, but can also be adapted to multiclass problems^35–37^.

#### Bagging

It is an ensemble learning method and is used to improve prediction performance. In this model, random samples are taken from the original dataset and an independent model is trained on each sample. Then, the predictions of all models are combined to form a prediction. Voting is used for classification problems and averaging for regression problems^38,39^.

#### Random forest

Random Forest is a powerful artificial intelligence algorithm that is one of the ensemble learning methods and combines a large number of decision trees. This algorithm is based on the bagging method and additionally uses feature randomization. Each decision tree is trained on a randomly selected sample from the original dataset and a randomly selected subset of features is used at each node^40^. This approach increases the diversity of the model and reduces the risk of overlearning. In the prediction phase, the results of all trees are combined. Majority voting is used for classification and averaging is used for regression. Random Forest has the advantages of high accuracy, good generalization ability and applicability to different types of data^26,41,42^.

## Experimental results

This section presents the results obtained from the experimental analysis of the machine learning algorithms used in the study. The process begins with a correlation matrix to explore the basic relationships between variables. Then, the classification performances of Logistic Regression, Bagging, and Random Forest algorithms are evaluated individually. Finally, all results are compared to identify which model performs best in predicting students’ coping strategies.

### Correlation matrix result

**Fig. 4:**
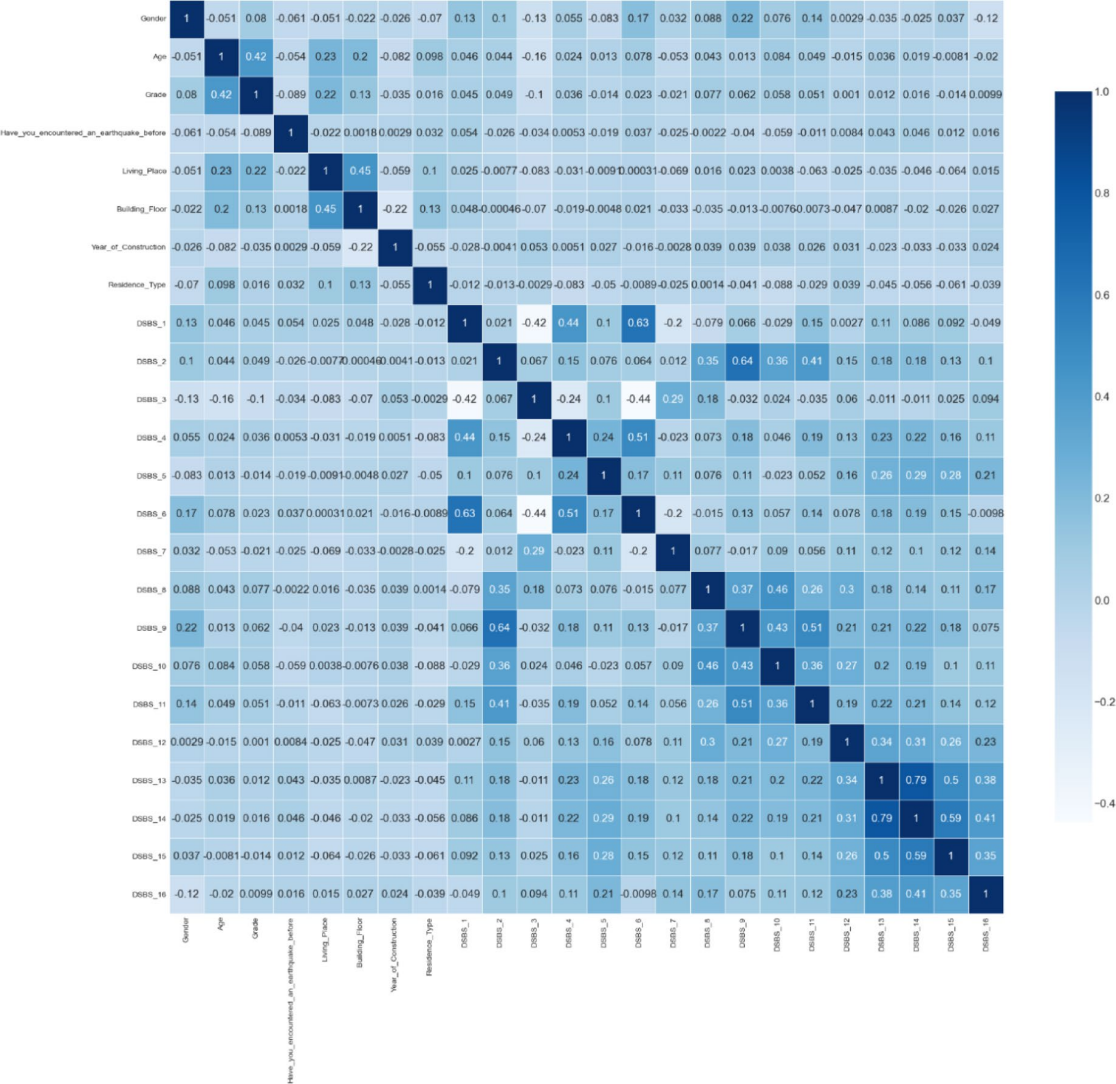
Correlation Matrix Result.

Figure 4 shows that there are significant correlations between the CESS items. In particular, there are high correlations (0.50 and above) between items such as CESS_13, CESS_14 and CESS_15. This may indicate that these classes may measure similar constructs or that participants responded similarly to these items.

On the other hand, the correlations of demographic variables such as age, gender and grade with CESS items are generally low. There is a negative correlation (−0.13) between gender and CESS_3. This may indicate that there is a significant, albeit weak, inverse relationship between gender and this item. In general, there is no significant correlation between demographic variables and CESS items. This may indicate that the effect of demographic characteristics on post-earthquake symptoms may be limited.

### Results of LR algorithms

The parameter values for the Logistic Regression algorithm are presented in Table 6. Looking at the results of the LR algorithm given in Table 7, it is seen that a total of 846 correct predictions, 103 “Disagree”, 388 “Agree” and 355 “Strongly Agree”, were made out of 858 data, while 12 incorrect predictions were made. All of the incorrect predictions made by the LR algorithm are between the “Disagree” and “Agree” classes. The LR algorithm achieves 98.60% accuracy, 98.60% precision, 98.60% recall and 98.60% F1-Score.

**Table 6:** The parameter values for the algorithms.

Models	Parameter	
LR	Batch Size	100
	Debug Mode	False
	Do Not Check Capabilities	False
	Do Not Standardize Attributes	False
	Maximum Iterations	−1
	Number of Decimal Places	4
	Ridge (Regularization Parameter)	1.0E-8
	Use Conjugate Gradient Descent	False
RF	Bag Size Percent	100
	Batch Size	100
	Break Ties Randomly	False
	Calculate Out-Of-Bag Error	False
	Compute Attribute Importance	False
	Debug Mode	False
	Do Not Check Capabilities	False
	Maximum Depth	0
	Number of Decimal Places	2
	Number of Execution Slots	1
	Number of Features	0
	Number of Iterations	100
	Output OOB Complexity Statistics	False
	Print Classifiers	False
	Seed	1
	Store OOB Predictions	False
Bagging	Bag Size Percent	100
	Batch Size	100
	Calculate Out-Of-Bag Error	False
	Classifier	REPTree -M 2 -V 0.001 -N 3 -S 1 -L −1 -I 0.0
	Debug Mode	False
	Do Not Check Capabilities	False
	Number of Decimal Places	2
	Number of Execution Slots	1
	Number of Iterations	10
	Output OOB Complexity Statistics	False
	Print Classifiers	False
	Represent Copies Using Weights	False
	Seed	1
	Store OOB Predictions	False
	Classifier Parameter	Meaning
	-M 2	Minimum number of instances per leaf
	-V 0.001	Minimum variance proportion
	-N 3	Number of folds for reduced-error pruning
	-S 1	Seed
	-L −1	Maximum tree depth
	-I 0.0	Minimum information gain

**Table 7:** Results of LR Algorithm.

	Predicted				Performance Metrics (%)	Disagree	Agree	Strongly Agree	Total
Actual	Bagging	Disagree	Agree	Strongly Agree	Accuracy	-	-	-	98.60
	Disagree	103	6	0	Precision	94.50	98.50	100.0	98.60
	Agree	6	388	0	Recall	94.50	98.50	100.0	98.60
	Strongly Agree	0	0	355	F1-Score	94.50	98.50	100.0	98.60
					MCC	93.70	97.2	100.0	97.90

### Results of bagging algorithm

The parameter values for the Bagging algorithm are presented in Table 6. Looking at the results of the Bagging algorithm given in Table 8, it is seen that out of 858 data, a total of 686 correct predictions (54 “Disagree”, 377 “Agree” and 295 “Strongly Agree”) and 172 incorrect predictions were made. Bagging algorithm makes 63 incorrect predictions between “Disagree” and “Agree” and 109 incorrect predictions between “Agree” and “Strongly Agree” classes. In addition, the Bagging Algorithm achieved 79.95% accuracy, 80.80% precision, 80.00% recall and 79.50% F1-Score. Errors primarily occur between ‘Agree’ and ‘Strongly Agree,’ suggesting fuzzy boundaries in moderate coping levels.

**Table 8:** Results of bagging Algorithm.

	Predicted				Performance Metrics (%)	Disagree	Agree	Strongly Agree	Total
Actual	Bagging	Disagree	Agree	Strongly Agree	Accuracy	-	-	-	79.95
	Disagree	54	55	0	Precision	87.10	74.60	85.80	80.80
	Agree	8	337	49	Recall	49.50	85.50	83.10	80.00
	Strongly Agree	0	60	295	F1-Score	63.20	79.70	84.40	79.50
					MCC	62.30	60.60	73.70	66.30

### Results of RF algorithm

The parameter values for the Random Forrest algorithm are presented in Table 6. Looking at the results of the RF algorithm given in Table 9, it is seen that out of 858 data, a total of 737 correct predictions (60 “Disagree”, 362 “Agree” and 315 “Strongly Agree”) were made while 121 incorrect predictions were made. RF algorithm makes 51 incorrect predictions between “Disagree” and “Agree” and 69 incorrect predictions between “Agree” and “Strongly Agree” classes. In addition, the RF algorithm achieved 85.89% accuracy, 86.80% precision, 85.90% recall and 85.50% F1-Score. Errors primarily occur between ‘Agree’ and ‘Strongly Agree,’ suggesting fuzzy boundaries in moderate coping levels.

**Table 9:** Results of RF Algorithm.

	Predicted				Performance Metrics (%)	Disagree	Agree	Strongly Agree	TOTAL
Actual	Bagging	Disagree	Agree	Strongly Agree	Accuracy	-	-	-	85.89
	Disagree	60	49	0	Precision	95.20	80.30	91.60	86.80
	Agree	3	362	29	Recall	55.00	91.90	88.70	85.90
	Strongly Agree	0	40	315	F1-Score	69.80	85.70	90.10	85.50
					MCC	62.30	60.60	73.70	66.30

**Fig. 5:**
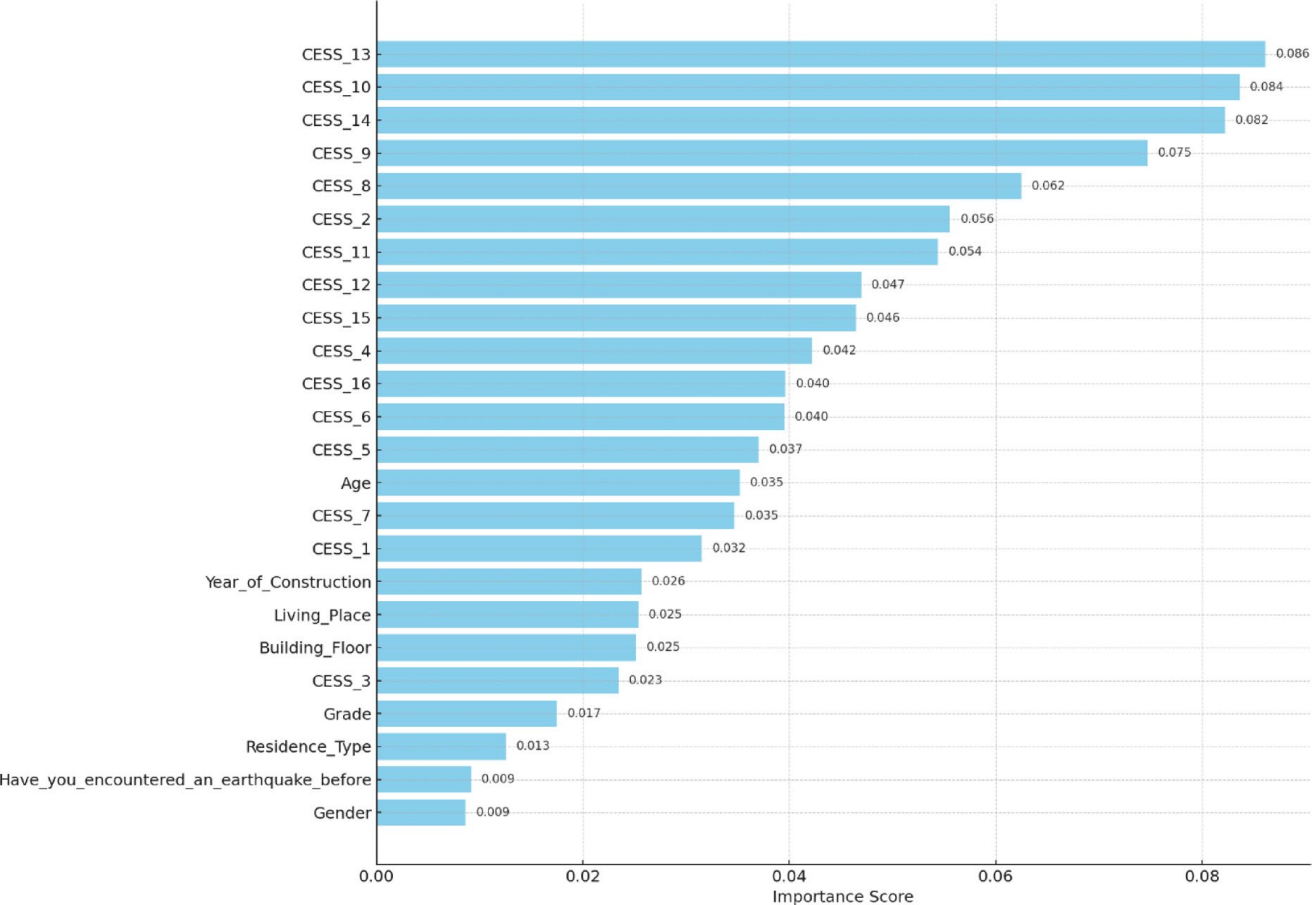
Feature Importances from Random Forest Model.

Feature importances from random forest model is shown in Fig. 5. Using the Random Forest algorithm, the contribution levels of variables to the model were determined. According to the calculated feature importance scores, the most influential variables were CESS_13 (0.086), CESS_10 (0.084), CESS_14 (0.082), and CESS_9 (0.075). These were followed by CESS_8 (0.062), CESS_2 (0.056), CESS_11 (0.054), and CESS_12 (0.047). Other variables with a moderate level of contribution to the model included Age (0.035), Year_of_Construction (0.026), Living_Place (0.025), and Building_Floor (0.025), which represent demographic and structural characteristics.

The variables with the lowest importance scores were Residence_Type (0.013), Have_you_encountered_an_earthquake_before (0.009), and Gender (0.009). This suggests that personal factors such as gender or prior earthquake experience had limited impact on the model’s predictive performance. Overall, the results indicate that the CESS-series variables made a significant contribution to the predictive capabilities of the model.

### Results of all classifications algorithms

Table 10 compares the performance metrics of three different artificial intelligence algorithms. Bagging algorithm performs lower than the other algorithms with 79.95% accuracy, 80.80% precision, 80.00% recall and 79.50% F-1 score. Random Forest performs better than Bagging with an accuracy of 85.89% and provides more balanced results with 86.80% precision, 85.90% recall and 85.50% F-1 score. Logistic Regression performs the best, outperforming the other algorithms with 98.60% accuracy, precision, recall and F-1 scores. The results show that the LR algorithm is the most successful method on this dataset, with RF coming in second place. Bagging, on the other hand, performs poorly compared to the other two algorithms. Additionally, Table 10 includes the majority-class baseline, which corresponds to always predicting the most dominant class in the dataset (‘Agree’). This baseline achieves an accuracy of only 45.92%, clearly showing that the proposed models (e.g., LR: 98.60% accuracy) perform significantly better than such a simplistic approach.

**Table 10:** Performance metric results of all Algorithms.

Algorithm	Accuracy (%)	Precision (%)	Recall (%)	F1-Score	MCC	Majority-class baseline (%)
Bagging	79.95	80.80	80.00	79.50	66.36	45.92
RF	85.89	86.80	85.90	85.50	76.70	
LR	98.60	98.60	98.60	98.60	97.90	

## Discussion and conclusion

This study, which was conducted to determine the coping strategies of the society with earthquake stress, was conducted using the “Coping with Earthquake Stress Scale (CESS) Dataset” dataset. Artificial intelligence algorithms were used on the dataset. LR, Bagging and RF algorithms were used to predict coping strategies with earthquake stress. The LR algorithm achieved the highest classification accuracy with 98.60%. The RF algorithm has a classification accuracy of 85.89% and the bagging algorithm has a classification accuracy of 79.95%. The fact that the metric values are close to the accuracy values in all algorithms shows that the algorithms have balanced performance. When the confusion matrix results are analyzed, it is seen that almost all classes are separated without confusion in the LR algorithm. In the RF algorithm, confusion between classes is rare, while in the bagging algorithm, confusion occurred in all 3 classes, but considering that this overall performance is around 80%, it is still a high class identification rate.

The results of this study demonstrate that artificial intelligence methods can be effectively used to predict individuals’ coping strategies following earthquakes, offering an innovative approach to the field of disaster psychology. While traditional psychological assessments largely rely on self-reports, AI-based models analyze patterns within survey responses to generate predictive classifications. This methodological innovation combines psychological evaluation with data-driven techniques, contributing to the literature and opening new avenues for more objective, scalable, and early-detection approaches in post-disaster mental health research.

The findings of this study can also be interpreted in the context of psychological constructs such as fate beliefs and optimism, which are known to influence individuals’ coping strategies in disaster situations. Previous research has shown that individuals with a strong belief in fate often adopt more emotion-focused or passive coping strategies, as they tend to perceive disasters like earthquakes as unavoidable events beyond personal control. Conversely, individuals with higher levels of optimism are more likely to engage in problem-focused coping and proactive preparedness behaviors, perceiving that their actions can make a meaningful difference in mitigating disaster-related stress.

In our dataset, the distribution of coping strategies may partially reflect these underlying tendencies. For instance, participants classified as adopting more adaptive coping strategies could be interpreted as having higher levels of optimism or lower fatalistic beliefs, whereas those in less adaptive clusters may align with a more fatalistic perspective. While the present study did not directly measure fate beliefs or optimism, the observed patterns suggest that integrating these psychological constructs into future models could enhance the interpretability of AI-based predictions and provide a more holistic understanding of post-disaster coping behavior.

Significant performance differences were observed among the classification algorithms used in this study. Notably, the Logistic Regression algorithm achieved higher accuracy on the test data compared to other algorithms. This may be attributed to the distinct class separations within the dataset and Logistic Regression’s linear discriminative structure, which aligns well with such patterns. Furthermore, the algorithm’s low variance and strong generalization capability make it less sensitive to noise and enable it to produce more stable results.

On the other hand, ensemble algorithms based on decision trees, such as Bagging and Random Forest, possess greater flexibility and capacity to handle complex data structures. However, this flexibility can also make them more sensitive to class overlap, imbalanced distributions, and noise within the dataset. Therefore, although these algorithms theoretically have the potential to build stronger models, their performance may vary depending on the nature of the data. In this context, model selection should consider not only overall accuracy but also factors such as the structural characteristics of the dataset, class distribution, and noise levels. Although MCC supports the results, the high LR accuracy may reflect dataset-specific separability; validation on external data is recommended to mitigate overfitting risks.

The results obtained indicate that artificial intelligence methods can be effectively utilized to predict individual coping tendencies following disasters. This opens the way for a novel methodological approach in disaster psychology and offers a significant alternative to traditional assessment methods. Class imbalance was not mitigated to retain data integrity, but alternatives like class weighting in LR/RF could enhance minority-class recall without synthetic alterations, improving applicability in disaster psychology.

### Limitations

This study has several limitations that should be acknowledged. First, the models were trained and evaluated using cross-validation only, without an independent external validation dataset. This may increase the risk of overfitting, particularly for models achieving very high accuracy, such as Logistic Regression. Second, the dataset contained class imbalances and ordinal survey data recategorized for classification, which may have caused information loss and class overlap, influencing some algorithms (e.g., Bagging). Third, the study relied entirely on self-reported data, which are inherently prone to response biases and subjectivity. Finally, the current findings are based on a single, cross-sectional dataset; thus, longitudinal validation is needed to confirm the stability and generalizability of the predictive models.

Future studies can address these limitations by (i) collecting larger and more demographically diverse samples, (ii) incorporating independent test sets, and (iii) adopting longitudinal designs to track coping strategies over time. These steps will improve the robustness and applicability of AI-based approaches to post-disaster psychological research.

### Future works

With this study, future applications can focus on testing different artificial intelligence algorithms in addition to existing algorithms and developing hybrid models. In addition, in future studies, the generalizability of the results can be increased by expanding the dataset with larger and more demographically diverse groups. In the current study, there were difficulties in predicting some classes, such as the Bagging algorithm not predicting the “Agree” class exactly. The ordinal data in the dataset used for this study were categorized by us specifically for the classification tasks. Since there are no sharp boundaries between the classes, alternative methods could also be employed for analysis. In future studies, this problem can be overcome by applying techniques to reduce data bias. As an extension of this study, longitudinal studies could be conducted to track the knowledge and preparedness of the same participants over time. This could help evaluate the impact of educational programs to increase earthquake awareness.

## Data Availability

The data used to support the findings of this study are available on a publicly accessible repository:https://nigmetkoklu.com/datasets/CESS_Dataset.zip. For any questions or further requests regarding the dataset, please contact Assist.Prof.Dr Suleyman Alpaslan Sulak at sulak@erbakan.edu.tr.
